# The Transferrin Receptor-Directed CAR for the Therapy of Hematologic Malignancies

**DOI:** 10.3389/fimmu.2021.652924

**Published:** 2021-03-29

**Authors:** Zilong Guo, Yirui Zhang, Mingpeng Fu, Liang Zhao, Zhen Wang, Zhuoshuo Xu, Huifen Zhu, Xiaoli Lan, Guanxin Shen, Yong He, Ping Lei

**Affiliations:** ^1^ Department of Immunology, School of Basic Medicine, Tongji Medical College, Huazhong University of Science and Technology, Wuhan, China; ^2^ Department of Nuclear Medicine and PET Center, Zhongnan Hospital of Wuhan University, Wuhan, China; ^3^ Department of Laboratory Medicine, Guangzhou First People’s Hospital, School of Medicine, South China University of Technology, Guangzhou, China; ^4^ Department of Nuclear Medicine, Union Hospital, Tongji Medical College, Huazhong University of Science and Technology, Wuhan, China

**Keywords:** transferrin receptor, chimeric antigen receptor, alternative target, hematological malignancies, T-cell acute lymphoblastic leukemia

## Abstract

As many patients ultimately relapse after chimeric antigen receptor (CAR) T-cell therapy, identification of alternative targets is currently being evaluated. Substantial research efforts are underway to develop new targets. The transferrin receptor (TfR) is prevalently expressed on rapidly proliferating tumor cells and holds the potential to be the alternative target. In order to investigate the efficacy and challenges of TfR-targeting on the CAR-based therapy strategy, we generated a TfR-specific CAR and established the TfR-CAR–modified T cells. To take the advantage of TfR being widely shared by multiple tumors, TfR-CAR T cells were assessed against several TfR^+^ hematological malignant cell lines. Data showed that TfR-CAR T cells were powerfully potent in killing all these types of cells *in vitro* and in killing T-ALL cells *in vivo*. These findings suggest that TfR could be a universal target to broaden and improve the therapeutic efficacy of CAR T cells and warrant further efforts to use these cells as an alternative CAR T cell product for the therapy of hematological malignancies.

## Introduction

As a prominent representative of immunotherapy, the chimeric antigen receptor (CAR)–modified T cells have made inspiring results in the clinical treatment of various tumors, especially for hematologic malignancies in this decade, which brought out bright to conquer cancer (1–4). The CAR generally incorporates extracellular tumor antigen recognition domain (such as ligand, scFv derived from immunoglobulin) and intracellular T-cell activating domains (5, 6). Hence, CAR T cells follow the mechanism of antibody combining specifically with antigen and thereby break the major histocompatibility complex (MHC) restriction to recognize tumor antigens directly (7, 8). To date, the preclinical study and clinical trial of CAR T cell therapy have made great strides long. However, it is still facing several challenges, in particular antigen-loss and antigen-low escape, to be dealt with (9, 10). Accordingly, there remains a need for developing a novel target to broaden and improve the therapeutic efficacy of CAR T cells.

Transferrin receptor (TfR or CD71), as a crucial route of cellular iron uptake, acts as a binding carrier of transferrin (TF) and transports the TF-iron complex to the cytoplasm *via* receptor-mediated endocytosis (11, 12). TfR is overexpressed on cancer cells attributing to the increased need for iron as a cofactor involved in DNA synthesis of rapidly dividing cancer cells (13). It plays an important role in tumor proliferation, invasion, and metastasis (14–16). And its overexpression has been associated with poor prognosis in cancer patients. Due to the notably elevated expression and its indispensable role in physiological and pathological processes of tumor cells, TfR is an attractive targeting molecule that could potentially be used to treat a variety of malignancies (17, 18). Experiments have validated the efficacy of various anti-TfR mAbs in inducing apoptosis of adult T-cell leukemia/lymphoma (19), erythroleukemia (20), and so on. Henry et al. reported that ^89^Zr-Tf by binding with TfR built a valuable tool to noninvasively assess oncogene status and target engagement of small-molecule inhibitors downstream of oncogenic KRAS (21). Clinical trials have being conducted to evaluate the safety and efficacy of agents targeting TfR in cancer patients with promising results (22). Our preceding researches also revealed that the therapeutic strategies around TfR enhanced the antitumor effects (23–25). And anti-TfR mAb connected to functionalized HPPS nanoparticle enabled amalgamation of therapy and diagnosis to TfR^+^ tumors (26). These optimized antibodies exhibited secure and efficacious anti-tumor activity and proved TfR was a worthwhile pharmaceutical target for the development of tumor therapy. With special focus on T cell–redirection strategies for cancer treatment, the anti-TfR mAb has been developed to generate TfR-targeted bispecific T-cell engager antibodies (27) and the TfR-BiTE was proven to have the ability to induce the selective lysis of various TfR^+^ cancer cells through the activation of T cells (28), assuring the application of TfR as target for this type of immunotherapies.

In order to investigate the efficacy and challenges of TfR-targeting on another T cell–redirection strategy, CAR-based therapy strategy, we generated a TfR-specific CAR and established the TfR-CAR–modified T cells. To take the advantage of TfR being widely shared by multiple tumors, TfR-CAR T cells were assessed against several hematological malignant cell lines. Data showed that TfR-CAR T cells were powerfully potent in killing all these types of cells *in vitro* and in killing T-ALL cells *in vivo*, suggesting TfR to be a universal target to broaden and improve the therapeutic efficacy of CAR-modified cells.

## Materials and Methods

### Cell Culture

U266, MOLT-4, KG­1a, K-562, HepG2 cell lines were preserved in our lab. Luc-MOLT-4 was established by engineering MOLT-4 cells with luciferase-expressing lentivirus (DesignGene Biotechnology, Shanghai, China). 293T (embryonic kidney cell) was obtained from the Cell Bank of the Chinese Academy of Sciences (Shanghai, China). Hematological malignant cell lines were maintained in RPMI1640 medium (Gibco; Thermo Fisher Scientific, Suzhou, China), supplemented with 10% fetal bovine serum (FBS) (Natocor, Córdoba, Argentina). HepG2 and 293T were maintained in DMEM medium (Gibco) supplement with 10% FBS.

Heparinized peripheral blood samples were obtained from healthy donors with approval from the Medical Ethics Committee of Tongji Medical College. Peripheral blood mononuclear cells (PBMC) were isolated by Ficoll density centrifugation and cultured in X-VIVO 15 Medium (Lonza, Walkersville, USA) supplemented with 5% FBS (Natocor).

### CAR Design and Viral Vector Production

*TfR-CAR* was generated by using commercial gene synthesis of an anti-TfR scFv previously reported by us (27, 28). The scFv was cloned into the backbone of a second-generation CAR with 4-1BB internal signaling domains in the pEF1α-T2A-EGFRt lentiviral vector. The construct was modified to express the truncated EGFR *via* a T2A peptide to enable detection of CAR following viral transduction. TfR-CAR lentiviruses were produced by transfecting 293T cells with the lentiviral packaging vector according to the standardized protocol (29). In brief, 293T cells were transfected with pEF1α-TfR CAR-T2A-EGFRt plasmids together with the packaging plasmids using polyethylenimine (Polysciences, Warrington, USA). The culture supernatants were harvested 24, 48, and 72 h later. Virus solutions were filtered through 0.45 µm filters (Millipore, Darmstadt, Germany) and concentrated by ultracentrifugation (HITACHI, Japan).

### Gene-Edited CAR T Cells

PBMCs were mixed with anti-CD3/CD28 Dynabeads (Life Technologies, Carlsbad, USA) at 4°C for 1 h. Then cells were separated using magnetic separator (Beaver Biomedical, Suzhou, China). The resuspended cells (1×10^6^ cells/ml) were further cultured in 24-well plates. After stimulated for 72 h, the medium was replaced with X-VIVO 15 serum-free medium. T cells were transduced with TfR-CAR lentivirus at an MOI of 25 with polybrene 6 µg/ml by centrifugation at 800*g* (relative centrifugal force (RCF)) for 90 min at 10°C. Transduced cells were expanded for 9 to 11 days in plates bound with anti-TfR mAb. Non-transduced T cells were treated the same and set as NC control. All cells were cultured in X-VIVO 15 Medium supplemented with hrIL-2 (30 IU/mL; Beijing Four Rings Biopharmaceutical, China) and 5% FBS.

### Western Blot Analysis

Whole cell lysates were separated by SDS-PAGE followed by blotting analysis with anti-CD247 (CD3ζ; Proteintech, Wuhan, China) and HRP-conjugated affinipure goat anti-rabbit IgG(H+L) (Proteintech). The protein band was developed using FDbio-Pico ECL kit (Fudbio science, Hangzhou, China) and photographed by the ChemiScope Imaging System (Clinx, Shanghai, China).

### Confocal Microscopy

The CFSE pre-stained HepG2 cells (TfR^+^) mounted onto sterile round coverslips were co-cultured with TfR-CAR T cells (or NC T cells) at 4°C for 90 min. After thorough washes, the mixed cell populations were fixed and stained with DAPI (Maravai LifeSciences, California, USA). Then the round coverslips were mounted onto glass slides with antifade reagent and observed using a confocal laser scanning microscope (Olympus, Tokyo, Japan).

### Activation and Degranulation Assay

0.5 to 1×10^5^ TfR^+^ malignant cells (U266, Molt4, Kg1a, and K562) were stained with Tag-it Violet™ tracking dye (BioLegend, San Diego, USA) followed by co-culturing with TfR-CAR T cells (E/T = 10:1) for 20 h. Then cells were harvested and measured for T-cell activation and degranulation indexes (perforin, granzyme B, FasL, CD25, and CD69) using flow cytometry.

### Cytotoxicity Assay

3×10^4^ Violet-prestained TfR^+^ malignant cells (U266, Molt4, Kg1a, and K562) were co-cultured with TfR-CAR T cells (or NC T cells) at various E/T ratios for 20 h. After transient centrifugation, the supernatants were analyzed for cytokines secretion, and cells were stained with 7-AAD (BioLegend) for cytotoxicity evaluation. The percentage of 7-AAD^+^ Violet^+^ cells was recorded by FCM to reflect cytotoxic activity of T cells.

### Cytokine Detection

The production of IL-2, IL-6, IFN-γ, and TNF-α were measured by the Cytometric Bead Array (CBA) Human Th1/Th2 Cytokine Kit II (BD Biosciences, San Jose, USA) according to the manufacturer's instructions. The results were analyzed using FCAP Array software version 3.0 (BD Biosciences).

### Flow Cytometry Analysis

The transduction efficiency of *TfR-CAR* in T cells was detected by direct staining with Alexa Fluor 647‐conjugated goat anti‐mouse IgG, F(ab′)_2_ fragment-specific antibody (Jackson Immunoresearch, West Grove, PA, USA), and by indirect staining using Cetuximab or TfR-huFc as the primary antibody followed by Alexa Fluor 647‐conjugated goat anti‐human IgG, Fc-specific antibody (Jackson Immunoresearch) as the secondary antibody. TfR expression on target cells was tested using anti-TfR mAb (purified by our lab) and PE-conjugated goat anti-mouse IgG (BD Biosciences). T cells were identified using FITC anti-huCD3 (HIT3a), APC-Cy7 anti-huCD4 (RPA-T4), BV510 anti-huCD8 (RPA-T8). The activation of T cells was evaluated using PE-Cy7 anti-huCD69 (FN50), APC anti-huFasL (NOK-1), PE anti-huPD1 (EH12.1), and PerCP-Cy5.5 anti-huCD25 (M-A251). The degranulation of T cells was evaluated using PE anti-huGranzyme B (GB11) and PerCP-Cy5.5 anti-huPerforin (δG9). The memory phenotypes were indicated using PE-Cy7 anti-huCD45RO (UCHL1), APC anti-huCD95 (DX2) and PerCP-Cy5.5 anti-huCCR7 (150503). For the analysis of Tregs populations, PE anti-huFoxp3 (259D/C7) and PerCP-Cy5.5 anti-huCD25 (M-A251) were used. Flow cytometry staining protocol has been previously described (28). Intracellular staining of granzyme B and perforin was performed using fixation/permeabilization solution kit (BD Biosciences). The nuclear transcription factor Foxp3 staining was performed using transcription factor buffer set (BD Biosciences). Unless otherwise stated, all antibodies were purchased from BD Biosciences. All samples were tested by the BD FACSVerse (BD Bioscience), and data were analyzed using FlowJo 10 software.

### T-ALL Mouse Xenograft Model

Six-week-old female NOD.Cg-Prkdc^scid^ Il2rg^tm1Vst^/Vst (NPG) mice (Beijing Vitalstar Biotechnology, Beijing, China) were used under a protocol approved by the Ethics Committee of Tongji Medical College of HUST. Mice were intravenously inoculated with 1 × 10^6^ Luc‐Molt4 cells. Once all mice demonstrated >1% huCD45^+^ cells in the peripheral blood, animals were randomly assigned to experimental treatment followed by 4 × 10^6^ T cells injection by tail vein and a second dosing 6 days later. Mice were weighed every other day; physical appearance and clinical signs, such as lethargy or persistent recumbency, body temperature, diarrhea, and paralysis, were monitored. Bioluminescent imaging (BLI) utilized the Tanon 5200 Multi-imaging system (Tanon Science & Technology Inc., Shanghai, China) with living image software (Tanon) for acquisition of imaging data sets. Luciferase activity was analyzed using ImageJ Software Version 1.8.0 and the photon flux analyzed within regions of tumor. If one tumor-bearing mouse exhibited the signs of morbidity (30), the experiments were terminated. All mice were sacrificed, and various organs were harvested for histopathologic analyses. Plasma cytokines were measured by CBA kit (BD Biosciences), plasma alanine aminotransferase (ALT) and aspartate aminotransferase (AST) were analyzed using corresponding assay kits (Nanjing Jiancheng Bio-engineering Institute, Nanjing, China). The activation and degranulation indexes, the memory T cell subsets, and the percentages of Tregs in the peripheral blood and spleen were analyzed as described above.

### Statistical Analysis

Percentage data were analyzed by Mann-Whitney U test using SPSS 17.0 statistical software (SPSS Inc., USA). Other data were analyzed by Student’s *t* test using GraphPad Prism 7 version (GraphPad Software, La Jolla, CA). Data were presented as means ± SD or SEM, as stated in the figure legends. *P* values< 0.05 were considered statistically significant.

## Results

### TfR-CAR T Cells Are Successfully Established

**Figure 1:**
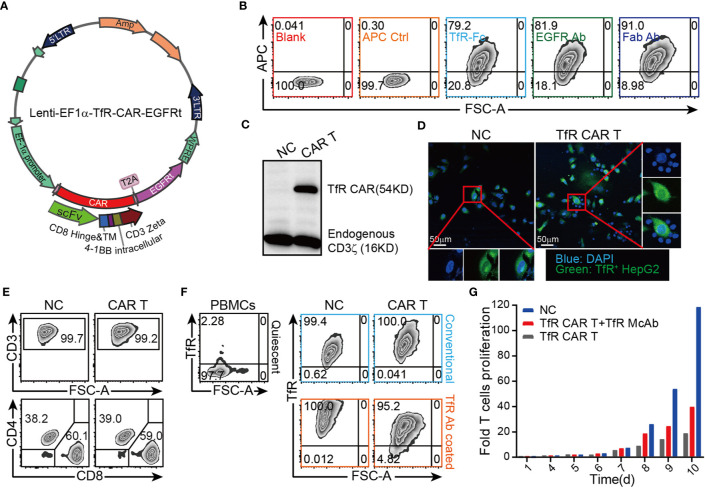
Design and establishment of TfR-directed CAR (TfR-CAR) T cells. **(A)** Schematic representation of the TfR-CAR lentivirus plasmid. The TfR-CAR expression cassette was under the regulation of EF1α promoter, and consisted of the hIL-2 signal peptide, anti-TfR scFv, the CD8 hinge and transmembrane region, and 4-1BB intracellular signaling domains, fused to the cytoplasmic region of the CD3ζ chain. A truncated human EGFR (EGFRt) transduction marker was separated with T2A. **(B)** FCM analysis showing TfR-CAR expression. TfR-CAR T cells were stained respectively with TfR-hFc, Cetuximab, anti-F(ab′)_2_ fragment. APC control described the CAR T cells were only stained with secondary antibody, but no TfR-Fc or Cetuximab. **(C)** Western blot analysis showing TfR-CAR expression. Whole cell lysates were hybridized with anti-hCD3ζ and HRP-conjugated goat anti-rabbit IgG. Molecular weight of the endogenous CD3ζ chain: 16 kDa; predicted molecular weight of the TfR-CAR: 54 kDa. **(D)** TfR-CAR T cells recognized and linked the TfR^+^ HepG2 cells. CFSE pre-stained HepG2 cells were incubated with TfR-CAR T cells or NC T cells for 90 min. Then cells were fixed and stained with DAPI. Rosette formation were detected by a confocal microscope. **(E)** The percentages of CD3^+^ cells and CD4^+^/CD8^+^ subsets in TfR-CAR T cells and NC T cells. **(F)** TfR expression on quiescent PBMCs and expanded T cells. Upper panel black signage: Quiescent PBMCs showed very low TfR expression. Upper panel blue signage: Expanded T cells cultured in conventional methods. Under panel orange signage: Expanded T cells cultured in anti-TfR mAb coated plates. **(G)** Expansion of TfR-CAR T cells. Transduced T cells were cultured in media supplemented with IL-2 (30 IU/mL) in plates coated with (red signage) or without (gray signage) anti-TfR mAb. Cell counts were recorded every day and fold cell expansion was calculated.

To evaluate the therapeutic potential of CAR T cells to recognize and kill tumors that express TfR, a TfR-based second-generation CAR, the anti-TfR scFv linked to the 4-1BB costimulatory domain and CD3ζ domain, was constructed and cloned in frame into a lentivirus vector (**Figure 1A**). Flow cytometric analysis showed that around 79.2% to 91.0% T cells were transduced with *CAR* gene and expressed the extracellularly localized scFv which could be detected by TfR-Fc fusion protein (79.2%) and murine F(ab′)_2_ antibody (81.9%). Anti-EGFRt tracking marker-specific staining (91.0%) also reported the expression of *CAR* on T cells. Moreover, the TfR-Fc-specific staining verified the ability of scFv to bind with the TfR target (**Figure 1B**). Western blot for CD3ζ in CAR-modified T cells (**Figure 1C**) exhibited a band with the expected molecular weight of 54 kDa, along with the band of 16 kDa endogenous CD3ζ. To investigate whether the CAR T cells could specifically recognize and bind with TfR^+^ cells, the suspension CAR T cells were co-cultured with adherent HepG2 cells (TfR^+^). If T cells engaged with HepG2 cells, they would also adhere to the culture surface and would not be removed by thorough washes. Immunofluorescence images showed that the CFSE-labeled HepG2 cells were surrounded by TfR-CAR T cells but not by NC T cells to form rosette-like configurations (**Figure 1D**). These results proved the recognizing and binding facility of CAR T cells to TfR^+^ tumor cells.

Our T cell expansion protocol generated CAR T groups with enrichment of CD8^+^ T cells and Tcm subsets, as well as the low percentage of Tregs (**Figure 1E**, **Supplementary Figures 3–4**). However, it was found that the expanded T cells greatly upregulated their expression of TfR which was expressed at a very low level in quiescent PBMCs (28) (**Figure 1F**, upper panel). To prevent suicide and fratricide to some degree, CAR-modified T cells were expanded in αTfR-coated plates, which blocked the TfR effectively abundance (**Figure 1F**, under panel). Even so, TfR-CAR T cells underwent a low rate of expansion (25-folds) compared to the NC group (up to 90-folds) within 10 days (**Figure 1G**).

### TfR-CAR T Cells Are Cytolytic to TfR^+^ Malignant Cells

**Figure 2:**
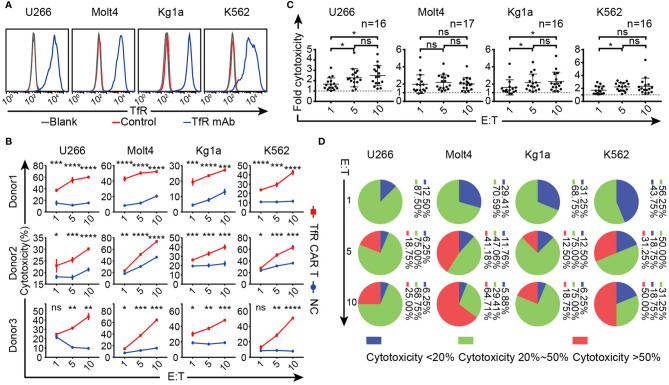
Therapeutic potency of the TfR-CAR T cells to TfR^+^ hematological malignant cells. **(A)** Expression of TfR on four hematological malignant cell lines. **(B–D)** TfR-CAR T cells were co-cultured with Tag-it Violet™-labeled malignant cells for 20 h at indicated E/T ratio, followed by staining with 7AAD. **(B)** The percentage of 7AAD^+^ Violet^+^ cells was recorded by FCM to reflect cytotoxic activity of T cells. Data from three representative donors are shown. **(C)** The fold of cytotoxicity of TfR-CAR T/NC T cells. Each dot represented a separate donor. n = 16–17. **(D)** The statistical interval distribution of cytotoxic activity of TfR-CAR T cells prepared from different individuals at indicated E/T ratios. Data are presented as means ± SD **(B)** or SEM **(C)**. *P < 0.05, **P < 0.01, ***P < 0.001, ****P < 0.0001, *ns* indicates no statistical differences.

The cytolytic capacity of CAR T cells was subsequently evaluated. After confirming the high TfR expression on several hematological malignant cell lines utilizing the anti-TfR mAb which contributed to scFv of CAR (**Figure 2A**), these cells were subjected to co-incubating with TfR-CAR T cells for cytotoxicity analysis. As shown in **Figure 2B**, 20 h co-culture made these cells vulnerable to CAR T cell-mediated cytolysis, and the lytic capacity increased in a E/T ratio-dependent manner. The response curves from 3 healthy donors also showed that TfR-CAR T cells from different individual sources exhibited different cytolytic capacity. Of these donors tested, 30% to 80% of malignant cells were lysed by TfR-CAR T cells (E/T = 10:1) within 20 h. **Figure 2C** confirmed that although the cytolytic activity of CAR T cells from different individuals varied, the cytolytic capacity of TfR-CAR T cells was several folds higher than those of NC T cells.

Furthermore, different malignant cell lines showed various sensitivities to CAR T cell-mediated cytolysis. At the same E/T ratio, Molt4 and K562 were more susceptible to the lysis than U266 and Kg1a. Although CAR T cells from different individuals exhibited different cytolytic capacity, when the E/T ratio reached 10:1, the killing rate of CAR T cells generated from 64.71% donors (11/17) against Molt4 exceeded 50% (**Figure 2D**).

Overall, the TfR-CAR T cells were capable of lysing TfR^+^ malignant cells, and T cell sources as well as tumor types were closely related to the CAR-T effectiveness.

### TfR-CAR T Cells Are Activated by Target Cells

**Figure 3:**
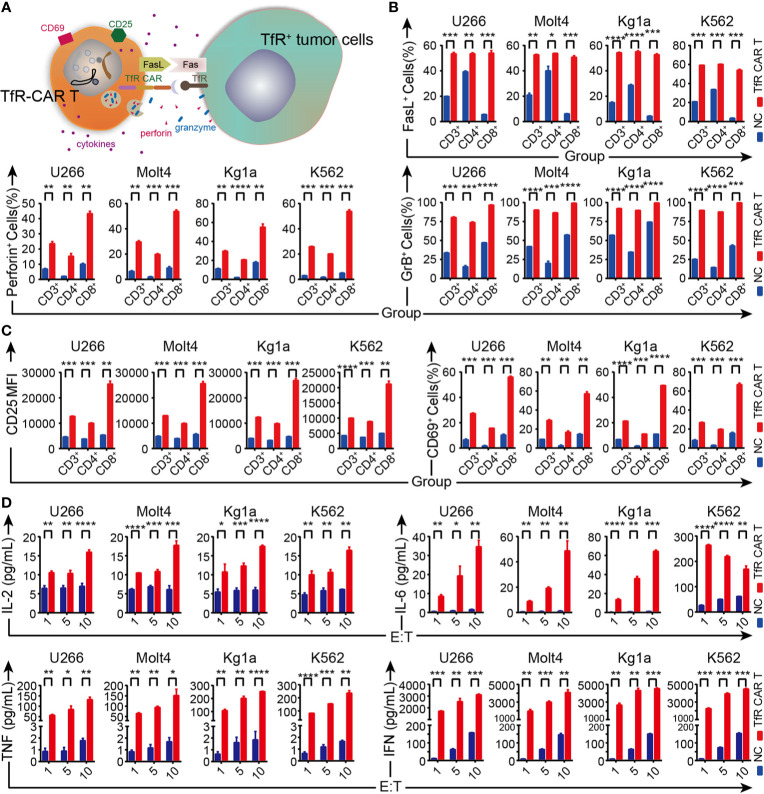
Analysis of TfR-CAR T cells degranulation, activation, and cytokine secretion capacity. **(A)** Schematic representation for the effector mechanisms of TfR-CAR T cells. CAR T-cells are activated upon recognition of TfR^+^ target cells, which resulted in upregulation of activation/cytotoxic markers on T cells, and production of pro-inflammatory cytokines. **(B, C)** TfR-CAR T cells and NC T cells were co-cultured with TfR^+^ hematological malignant cells (E/T ratio = 10:1) for 20 h. The expression of the cytotoxic markers FasL, perforin, and granzyme B were determined by FCM in gated CD3^+^, CD4^+^ and CD8^+^ T cell populations **(B)**, as well as the activation markers CD25 and CD69 **(C)**. **(D)** Cytokines released by T cells at indicated E/T ratios were detected by cytometric bead assay. Results are representative of three independent experiments performed with CAR T cells derived from three different donors. All data are presented as means ± SD. *P < 0.05, **P < 0.01, ***P < 0.001, ****P < 0.0001.

Previous studies have indicated that the perforin and granzyme release, Fas-FasL interactions were considered the main mechanisms by which activated CAR T cells can cause tumor cell lysis (31–33) (**Figure 3A** **)**. We thus examined FasL, intracellular perforin, and granzyme B expression on the T cells (including CD4^+^ and CD8^+^ subsets) when co-cultured with their target cells to assess their cellular cytotoxic potential. FCM analysis showed the expression of FasL as well as the intracellular granules was considerably upregulated on both CD4^+^ and CD8^+^ CAR T cells, although the up-regulation in CD8^+^ subsets was more pronounced than in the CD4^+^ subsets (**Figure 3B** **)**. Activation markers CD69 and CD25 displayed the same tendency on CAR T cells (**Figure 3C**). Moreover, the cytokines released by TfR-CAR T cells, including IL-2, IL-6, especially IFN, and TNF, were a lot more active than by NC T cells (**Figure 3D**).

Collectively, upon target cell stimulation, TfR-CAR T cells not only expressed higher levels of CD25, CD69, and FasL and showed a higher capacity for degranulation, but also more effectively mediated cytokine secretion, thereby exhibiting enhanced cytotoxicity to TfR^+^ malignant cells.

### TfR-CAR T Cells Are Potent in Killing T-ALL Cells In Vivo

**Figure 4:**
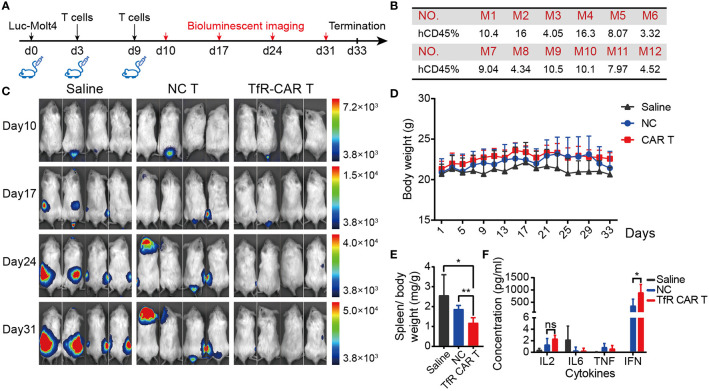
TfR-CAR T cells prevented T-ALL tumor progression *in vivo*. NPG mice were intravenously inoculated with Luc‐Molt4 cells (1 × 10^6^) on day 0. T cells (4 × 10^6^) were adoptively transferred on day 3 and day 9. Tumor burden were monitored by bioluminescent imaging on day 10, day 17, day 24, and day 31. If one tumor-bearing mouse exhibited the signs of morbidity, the experiments were terminated. Mice were sacrificed for main tissues and blood collection. **(A)** Schematic diagram for the development of xenograft mouse model. **(B)** Percentage of huCD45^+^ cells in the peripheral blood 48 h after engraftment. **(C)** Mice demonstrated >1% human ALL were randomly assigned to experimental treatment (n = 4 mice per cohort). Tumor progression were detected by *in vivo* bioluminescence imaging. Representative images of two independent experiments are shown. **(D)** Body weight and **(E)** Spleen weight over total body weight were analyzed. **(F)** Plasma cytokines were determined by CBA. All data are presented as means ± SD. *P < 0.05, **P < 0.01. *ns* indicates no statistical differences.

To characterize the TfR-CAR T cells in the setting of hematological malignancies, we next test their potency against T-ALL cell line Molt4 *in vivo*. For this purpose, immunodeficient NPG mice were xenografted with Luc-Molt4 cells, followed by a set of experiments as shown in **Figure 4A**. Well-established intravenous xenograft model was supported by FCM analyses of peripheral blood for huCD45. **Figure 4B** showed that 2 days after the inoculation of Luc-Molt4, huCD45^+^ cells in peripheral blood of all mice exceeded 1%. These mice were then randomly assigned to experimental treatment. Bioluminescent imaging demonstrated mice receiving TfR-CAR T cells were significantly protected from rapid tumor progression. The mice receiving NC T cells or saline, by contrast, exhibited malignant progression and had a higher degree of leukemia burden (**Figure 4C**, **Supplement Figure 7**). Overall, the saline group had the slowest weight gain, followed by the NC T group (**Figure 4D**). In particular, mice with metastatic brain tumors in NC T group had most significant weight loss. In contrast, the TfR-CAR T groups significantly reduced spleen weight (**Figure 4E** **)**. Analysis for plasma cytokine showed that TfR-CAR T treatment caused a considerable increase in IFN, as well as a trend toward increased IL-2 levels compared to NC T, although not statistically significant. The expression of IL-6, which played an important role in cytokine storms, was extremely low in both NC T and TfR-CAR T groups (**Figure 4F**).

**Figure 5:**
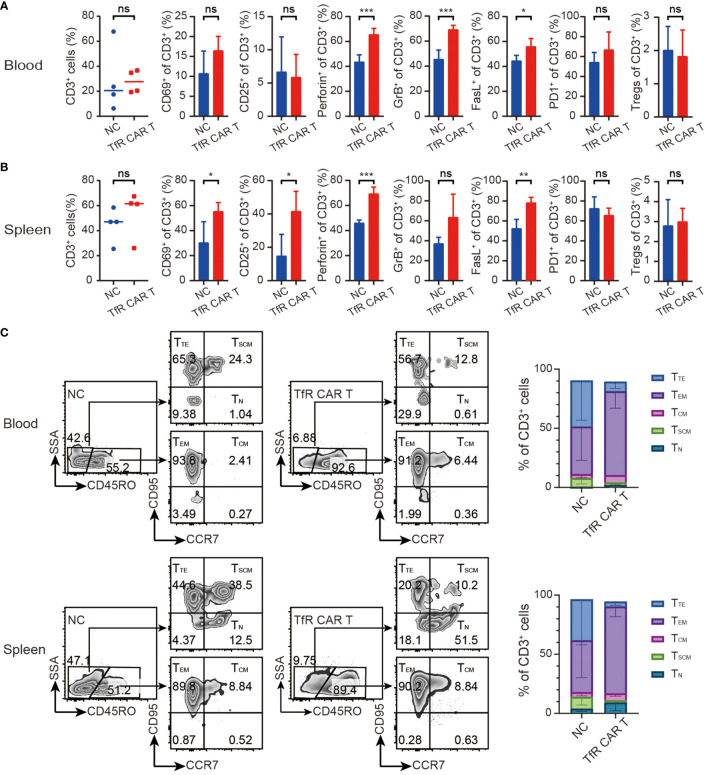
Characteristics of *in vivo* TfR-CAR T cells. Percentages and status of transferred human T cells in blood **(A)** and spleen **(B)** were analyzed by FCM. Tregs were defined as CD4^+^CD25^+^Fop3^+^ of CD3^+^ cells. All data are presented as means ± SD. *P < 0.05, **P < 0.01, ***P < 0.001, *ns* indicates no statistical differences. **(C)** Percentages of T_TE_ (Terminal effector, CD45RO^−^CCR7^−^CD95^+^), T_EM_ (Effector memory, CD45RO^+^CCR7^−^CD95^+^), T_CM_ (Central memory, CD45RO^+^CCR7^+^CD95^+^), T_SCM_ (Stem cell memory, CD45RO^−^CCR7^+^CD95^+^), and T_N_ (naïve, CD45RO^−^CCR7^+^CD95^−^) subtypes in CD3^+^ populations were measured by FCM. Representative FCM images (left) and stacked bar graphs (right) were shown. *n* = 4 per cohort.

To detect the presence and status of our transferred CAR T cells at termination day, transferred human T cells were monitored. As shown in **Figures 5A, B**, infused T cells were still detectable in mice peripheral blood and spleen 24 days after the final dosing of T cells. CAR T cells exhibited in the clear tendency of increasing CD69 and perforin, granzyme B, FasL expression when compared with NC T cells. Frequencies of PD1^+^ T cells and CD4^+^CD25^+^Fop3^+^ Treg showed no significant difference between the two groups. Moreover, the phenotype analysis of T cells differentiation showed that majority of the T cells from the TfR-CAR T group were prone to differentiation into T_EM_ (**Figure 5C**).

**Figure 6:**
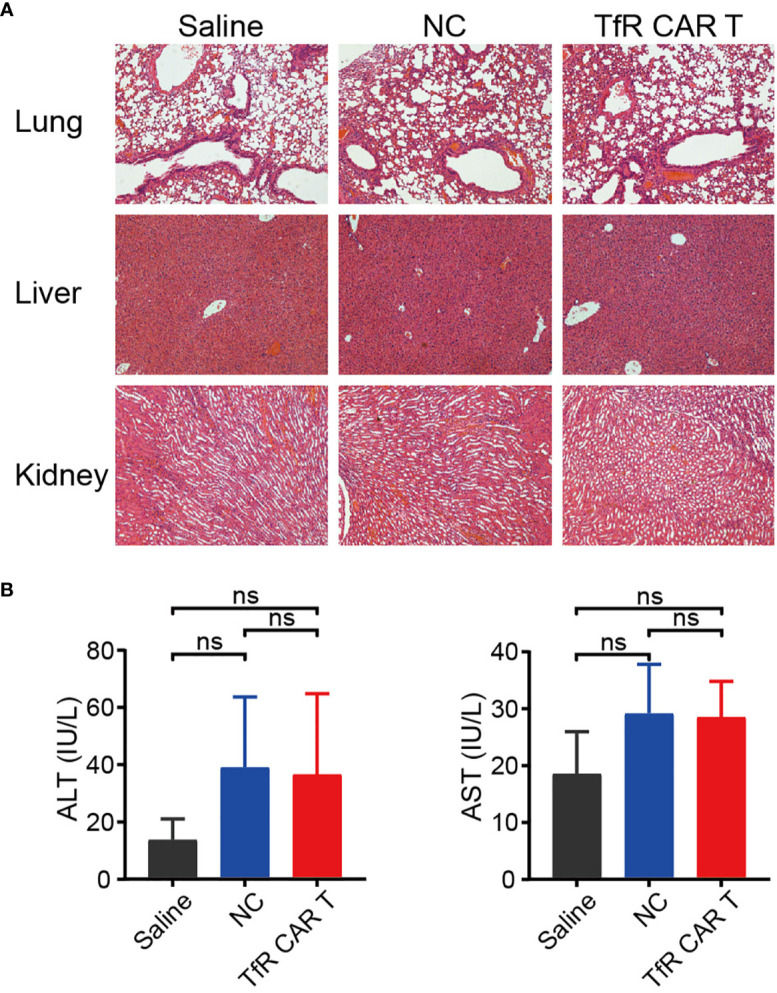
The safety evaluation of TfR-CAR T cell therapy. **(A)** H&E staining of the major tissues, including lung, liver, and kidney. Images were captured under 100× magnification. **(B)** Levels of plasma AST and ALT. *n* = 4 per cohort. All data are presented as means ± SD. *ns* indicates no statistical differences.

Finally, the biological safety of this product was evaluated. H&E staining showed that, following Luc-Molt4 and CAR T cells infusion, the mice did not develop a progressively worsening disease characterized by significant pathological changes in the major organs, including lung, liver, and kidney (**Figure 6A**). However, the levels of ALT and AST tended to increase slightly in mice treated with CAR T cells or NC T cells compared to those with saline, although not statistically significant (**Figure 6B**).

Collectively, these results suggested that TfR-CAR T cells were potent in killing T-ALL cells and elicited no systemic toxicity to xenografted mice.

## Discussion

While CAR-T therapy has achieved dramatic results in clinical trials for the treatment of hematological malignancies, some problems need to be addressed, especially antigen loss and antigen-low escape after treatment (9, 10). Therefore, it is necessary to identify more tumor-associated antigens as new therapeutic targets to develop CAR-T clinical application. As a gatekeeper regulating iron uptake, TfR is prevalently expressed on rapidly proliferating cells, like multiple types of tumors, making TfR a potential and valuable target for generating CAR-T cells. In this study, we designed a second-generation *TfR-CAR* to transduce T cells and performed the research of the anti-tumor effect *in vitro* and *in vivo*. The results showed that TfR-CAR T cells exhibited potent effector functions against four types of TfR^+^ hematological malignant cells *in vitro*, including specific recognizing and killing, degranulation, and robust cytokine production. Among the four hematological malignant cell types, T-cell acute lymphoblastic leukemia (T-ALL) cell line Molt4 was the most susceptible to TfR-CAR T cell-mediated cytolysis, followed by chronic myeloid leukemia cell line K562, myeloma cell line U266 and acute myeloid leukemia cell line Kg1a, although TfR expression level was comparable among these cell types. This tumor type-specific sensitivity to TfR-CAR T cells is worthy of our further investigation. The above results suggested that TfR-CAR T activity was TfR-specific, and the potency depended on TfR expression. Furthermore, TfR may have the advantage of being widely shared by multiple tumors, allowing for universal, off-the-shelf therapies.

Many patients with B-cell malignancies have achieved complete remission after receiving CAR T cells targeting the pan-B-cell antigen CD19 (34). The success of CAR T cells needs to be broadened to T-cell malignancies. Pan T-cell antigens CD3, CD5 and CD7 have been exploited to direct CAR-T cells against T-cell malignancies (35–37). However, the ability to target more than one T cell-associated antigen may be critical for the effective long-term treatment of malignancies arising from these cells. Hence, Molt4 (T-ALL cell line) xenografted mice were administered TfR-CAR T cells and found that this therapy produced substantial cytotoxicity against the T-ALL line and restrained malignant progression *in vivo*. These results established the feasibility of using TfR-CAR T cells for the targeted therapy of T-cell malignancies.

However, the application of TfR as a target for CAR T cell therapy in T-cell malignancies still faces the difficult problem met by many targetable antigens which are shared between normal and malignant T cells, resulting in the mutual killing of CAR T cells to greatly reduce their therapeutic efficacy (38). Our data and previous reports show that the TfR expression on quiescent PBMCs is considerably low (28) but has a certain upregulation on activated T cells. Persis J Amrolia et al. also reported that up to 39.3% T cells (basal line 2.6%) upregulated their TfR expression within 3 days of mixed lymphocyte reaction (39), but this figure rose to 65% ± 23% on proliferating alloreactive T cells. In this research, our CD3/CD28 Dynabeads-expanded T cells were found to substantially elevate their TfR expression. Since the generation of CAR-T cells must undergo activation by CD3/CD28 dynabeads, the application of the TfR-CAR T cells would deal with the challenges of how to avoid the mutual killing of themselves. Given the indispensability of TfR in cellular physiological activities, it is not advisable to use gene editing techniques to knock out TfR expression on T cells as genomic disruption of the CD7 gene in CD7-directed CAR T cells (36). In this study, we took the approach of culturing CAR-T cells in anti-TfR antibody-coated plates. These parental mAbs could partially block the scFv of TfR-CAR T cell to bind with targets on itself or cells in the vicinity. This try rescued TfR-CAR T cells from heavily impaired expansion to a considerable expansion (25-folds). To better address this question, other tries were proposed to circumvent CAR T cells fratricide. Wendell A. Lim reported a small molecule-gated ON-switch CAR which allowed CAR-T cells to remain silencing their therapeutic functions in the absence of an activating small molecule during the T-cell expansion phase *in vitro* (40). Michel Sadelain integrated combinatorial antigen recognition to render CAR-T cells destroy tumors only when the target cells expressed both antigens (41). In addition, another promising approach is transducing CARs into non-T cells, like NK cells. CAR NK cells directed against CD3 (42), CD4 (43), and CD5 (44) have been researched for killing T-cell malignancies and demonstrated potent anti-tumor activity *in vivo* and *in vitro*. This inspired us to use AND-gated circuits to generate a dual CAR or establish TfR-CAR NK to maximize TfR-directed CAR cell activity.

In our research, it was found that NC cells were still present even after several days of cell transfer. On this issue, inconsistent results have been observed across the literature. Reports showed that T cells could persist for a relatively long time after T cells infusion (45–47). On the contrary, Li et al. demonstrated that CAR-T cells could exist only for 5 to 7 days after CAR-T cells transfusion (48). The T cells in these independent studies were not manufactured *via* the same process, and the isolation, activation, culture conditions, and even expansion time *in vitro* affected the viability and development stages of the T cells. Furthermore, T cells were infused in two batches in this paper, and the termination point is relatively early. These may also result in the presence of NC cells several days after cell transfer. It was also found NC T cell also had a certain killing ability against malignant cells when compared with saline control. And the CAR T cells generated from individuals with strong NC T cell cytotoxicity would consequently have a stronger killing ability, suggesting that the therapeutic efficacy of CAR T cells is closely related to the patient’s immunocytes function itself and can be further improved by detecting and assessing the patient’s immune cell status in advance. This, in combination with the tumor type-specific sensitivity to TfR-CAR T cells, suggested that immune cell status-targets-indications fit has a significant influence on the effectiveness of CAR T therapy.

Our preliminary results showed that the timing and quantity of CAR T cell treatment had a significant impact on the anti-tumor effect, suggesting that the earlier the intervention, the higher the dose, the better the anti-tumor effect. This supported the conclusion that preconditioning chemotherapy to reduce tumor load was able to improve the efficacy of CAR-T cell therapy in clinical trials and suggested that repeated CAR T cell infusion could be administered for better efficacy with the premise of safety (49).

Although the TfR-CAR T cells showed potential cytotoxicity to Molt4 cells and controlled tumor progression *in vivo*, the treatment did not completely eradicate the burden of tumors. It may attribute to increased expression of inhibitory receptors, e.g., PD1, declining effector function of CAR-T cells (50). On the other, it could be that Molt4 cells have already formed a solid mass in the local tissues which is a well-documented clinical encounter for T cell leukemia (51, 52), making it difficult for CAR T to infiltrate in the mass and then mount an effective response. Therefore, it is quite important to overcome T cell exhaustion and increase CAR-T cell trafficking to the tumor mass to exert stronger therapeutic potency.

In conclusion, our study demonstrates the feasibility and efficacy of TfR-directed CAR T cell for the targeted therapy of TfR^+^ hematological malignancies, especially T-cell acute lymphoblastic leukemia. These findings suggest TfR be a universal target to broaden and improve the therapeutic efficacy of CAR T cells and warrant further efforts to use these cells as an alternative CAR T cell product for the therapy of hematological malignancies.

## Data Availability Statement

The original contributions presented in the study are included in the article/**Supplementary Material**. Further inquiries can be directed to the corresponding authors.

## Ethics Statement

The animal study was reviewed and approved by The Ethics Committee of Tongji Medical College of HUST.

## Author Contributions

ZG and YZ under the direction of PL, GS, and YH designed and performed the experiments, analyzed the data, and wrote the first version of the manuscript. MF, LZ, ZW, ZX, and HZ provided technical assists. XL discussed the manuscript and provided feedback and suggestions. PL and YH designed the experiments, revised the manuscript, and supervised the study. All authors contributed to the article and approved the submitted version.

## Funding

This research was funded by grants from the key program of the National Natural Science Foundation of China (Grant No. 82030052), the general program of NSFC (Grant No. 31570937, 81871391), and the Improvement Project for Theranostic ability on Difficulty miscellaneous disease (Tumor) (No. ZLYNXM202007).

## Conflict of Interest

The authors declare that the research was conducted in the absence of any commercial or financial relationships that could be construed as a potential conflict of interest
