# Supplementary material for: The Transferrin Receptor-Directed CAR for the Therapy of Hematologic Malignancies
Source: Front Immunol. 2021 Mar 29;12:652924. doi: 10.3389/fimmu.2021.652924 (PMC8039461; doi:10.3389/fimmu.2021.652924)
Supplement: Supplementary file 1 [file DataSheet_1.docx]

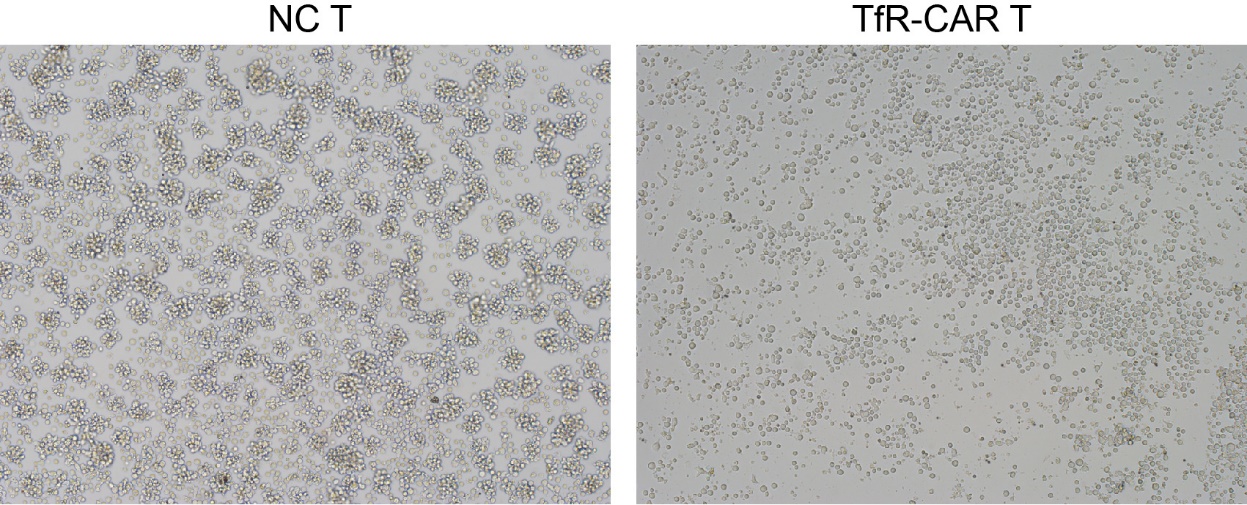


| Figure 1. Comparison of cell morphology of CAR T cell and NC T cell |
| --- |


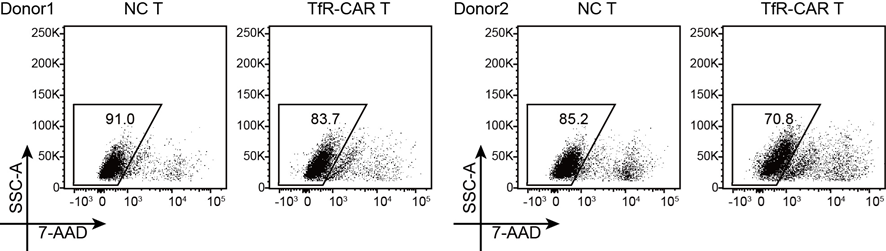


| Figure 2. Comparison of cell viability of CAR T cell and NC T cell |
| --- |


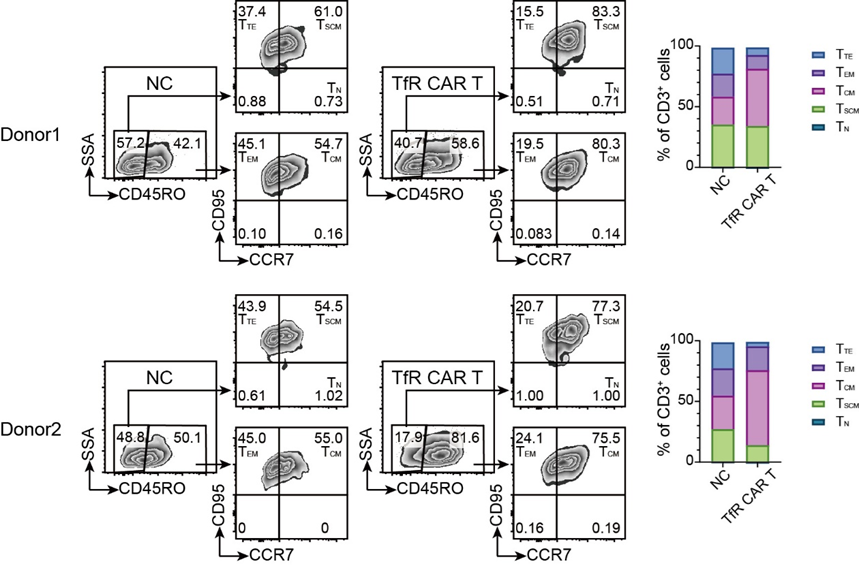


| Figure 3. Memory phenotype analysis of T cells |
| --- |


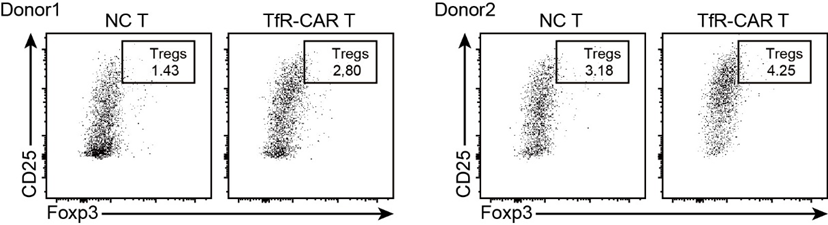


| Figure 4. The proportion of Treg cell population in T cells |
| --- |


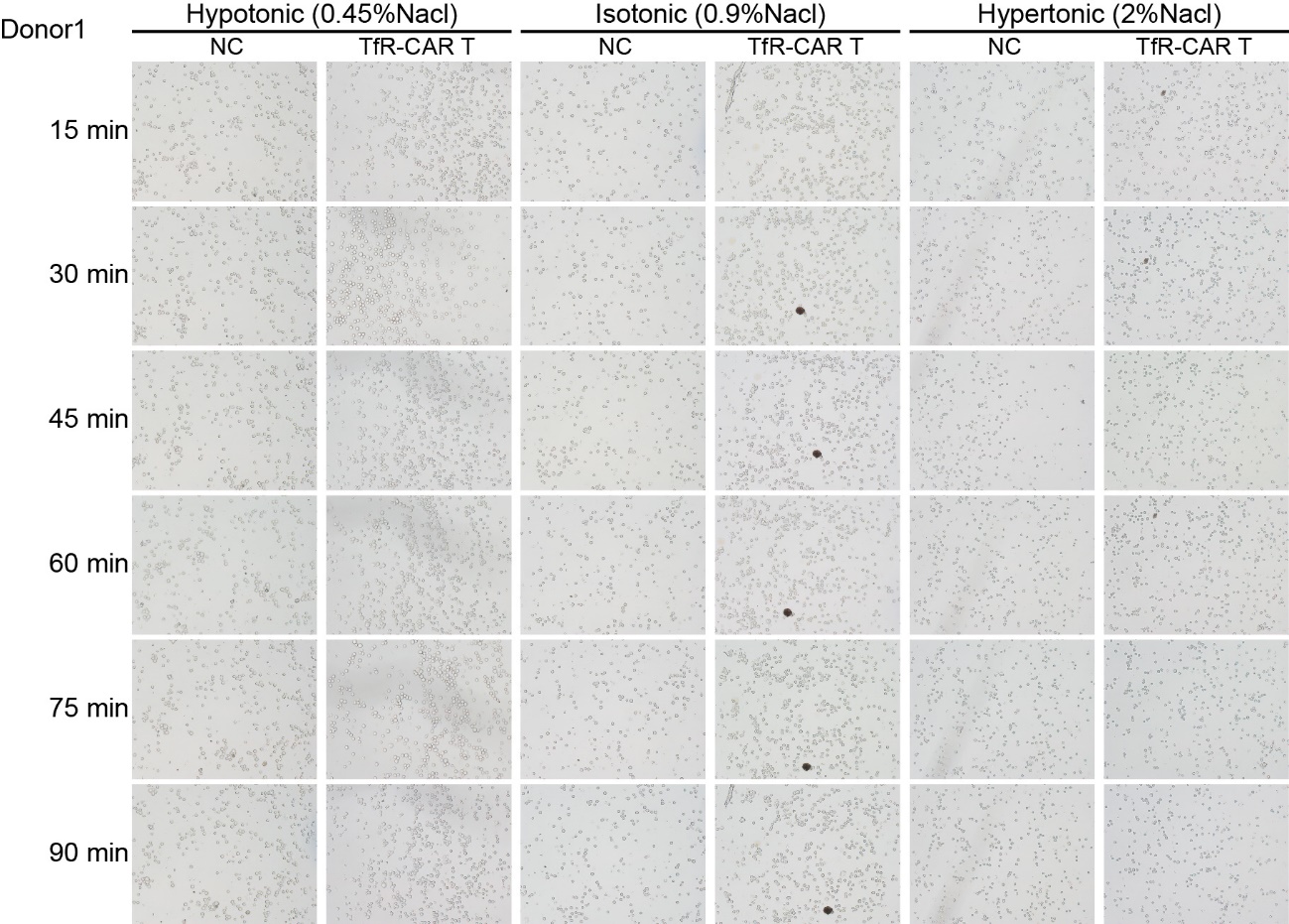


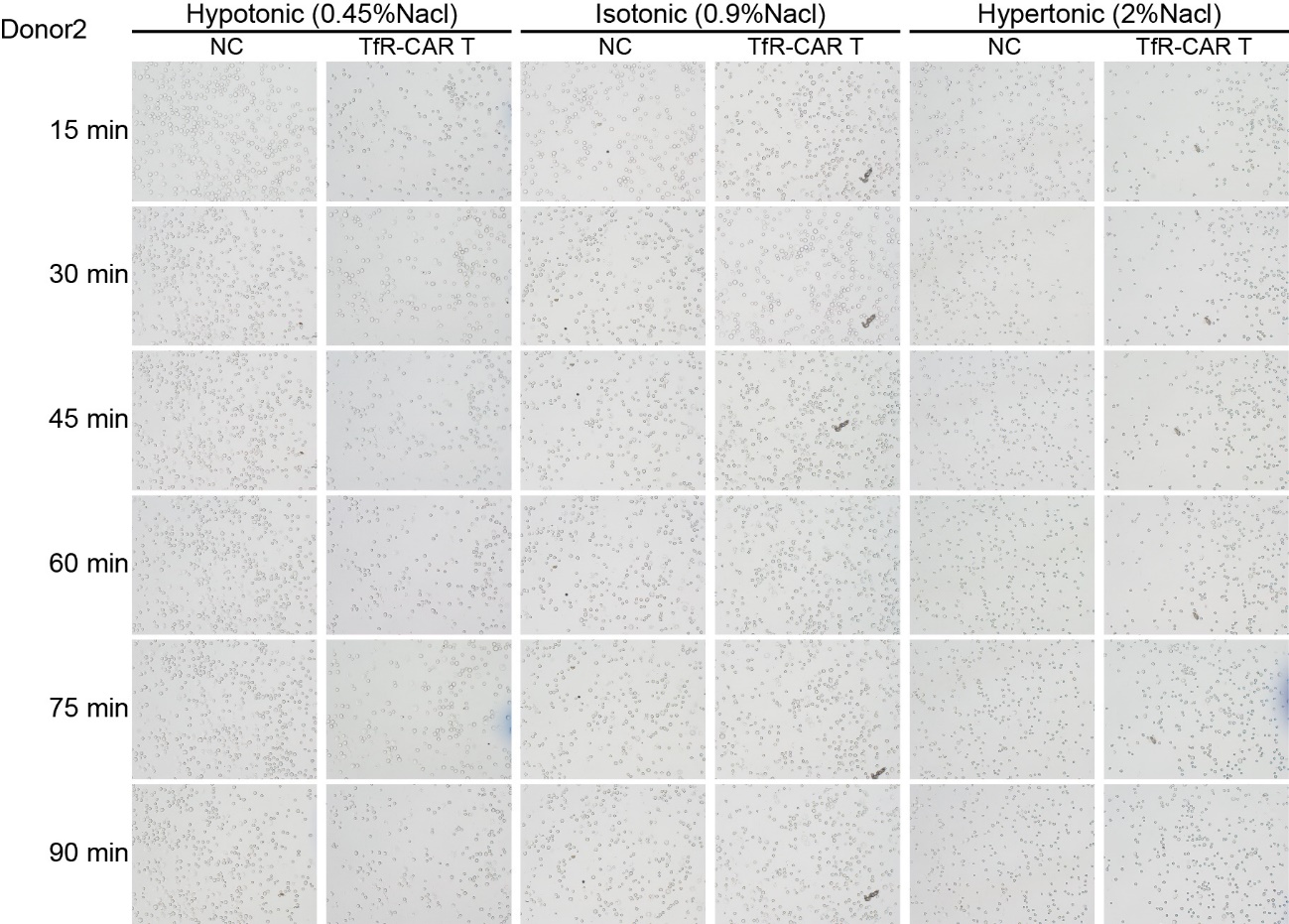


| Figure 5. T cells tonicity evaluation. T cells grown under hypotonic (0.45% NaCl), isotonic (0.9% NaCl), or hypertonic conditions (2% NaCl), were observed under a light microscope. Data from 2 representative donors are shown. |
| --- |


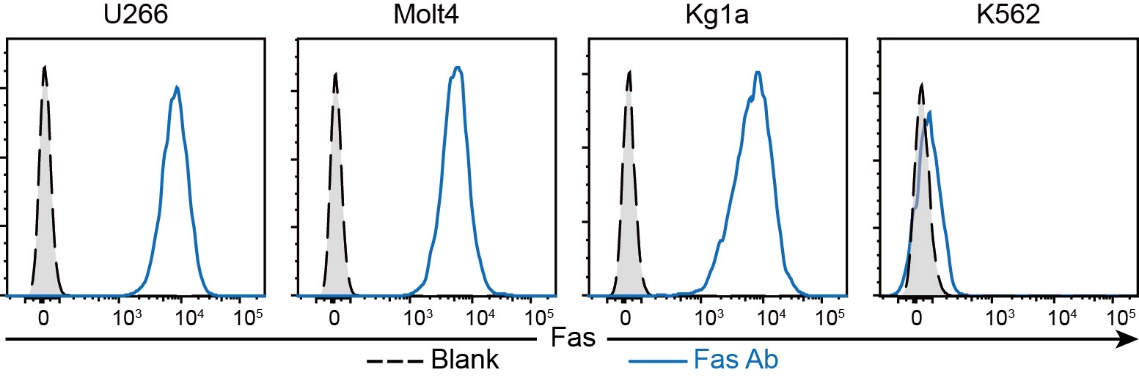


| Figure 6. Expression of Fas on four hematological malignant cell lines |
| --- |


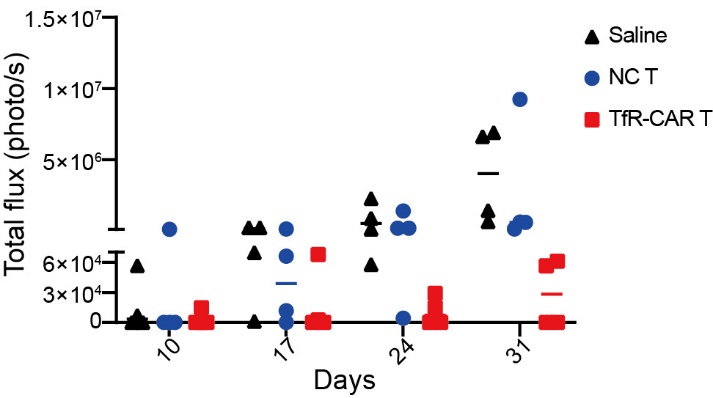


| Figure 7. Quantitative bioluminescence imaging |
| --- |


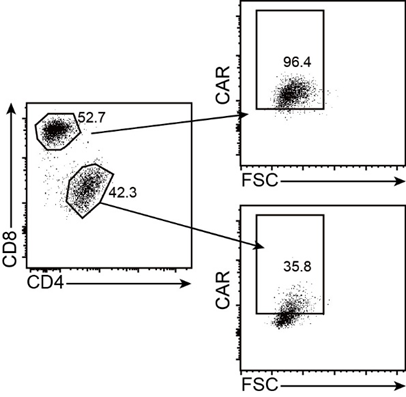


| Figure 8. Comparison of transduction efficiency of CAR in CD4 and CD8 subsets |
| --- |


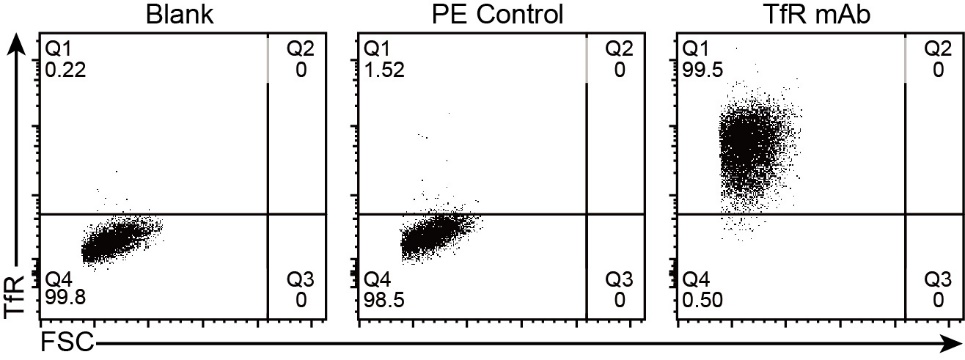


| Figure 9. TfR expression in HepG2 was assayed by flow cytometry |
| --- |


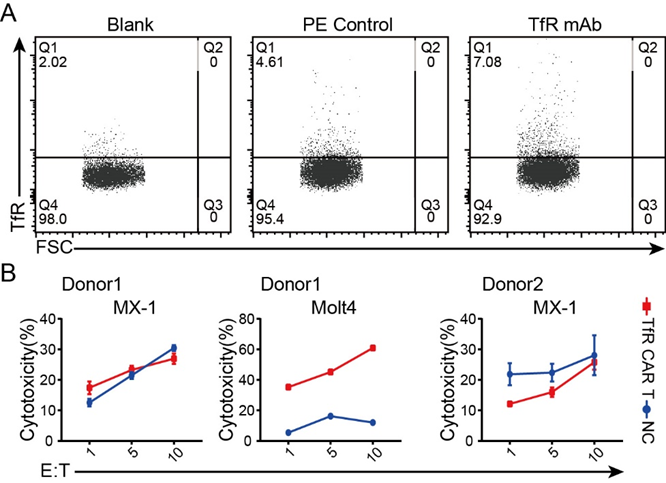


| Figure 10. Cytotoxicity assessment of TfR-CAR T cells against TfR-negative tumor cell line. (A) TfR expression in MX-1 was assayed by flow cytometry. (B) The percentage of 7AAD^+^ Violet^+^ cells was recorded by FCM to reflect cytotoxic activity of T cells. Data from 2 representative donors are shown. TfR^+^ tumor cell line Molt4 was used as a positive control. |
| --- |

|  |
| --- |


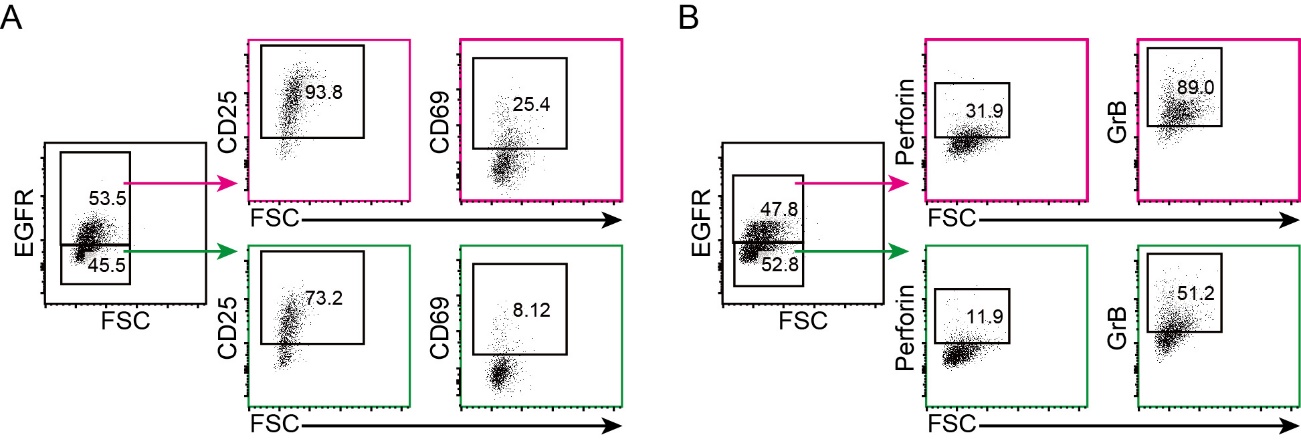


| Figure11. Comparison of activation and degranulation markers in EGFR^+^ and EGFR^-^ subsets of CAR T cell |
| --- |


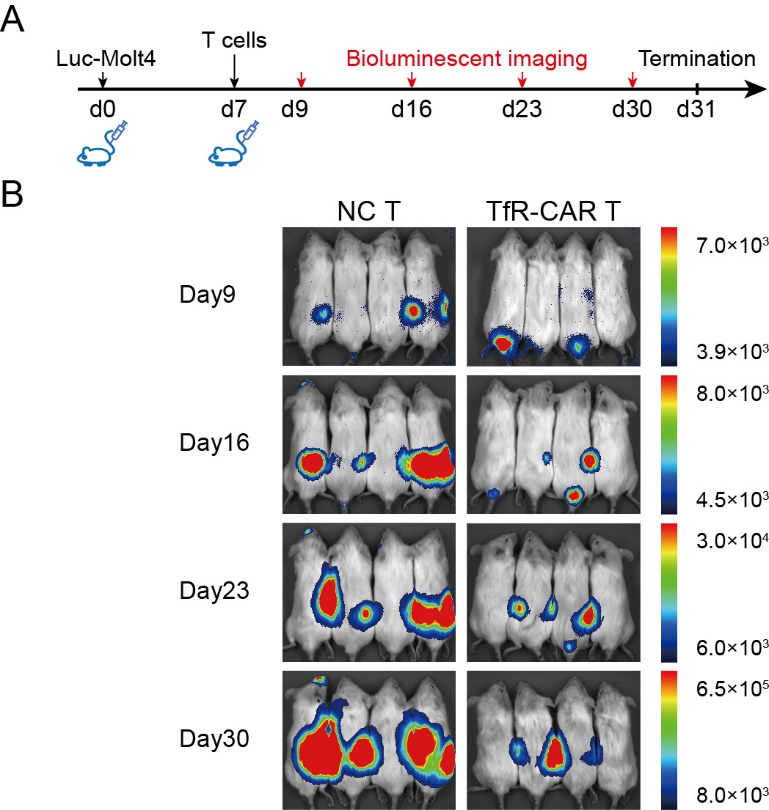


| Figure 12. Limited therapeutic effect on tumor-bearing mice with only one CAR T cell administration. NPG mice were intravenously inoculated with Luc-Molt4 cells (1×10^6^) on day 0. T cells (4×10^6^) were adoptively transferred on day 7. Tumor burden were monitored by bioluminescent imaging on day 9, day 16, day 23, and day 30. (A) Schematic diagram for the development of xenograft mouse model. (B) Tumor progression were detected by in vivo bioluminescence imaging. |
| --- |


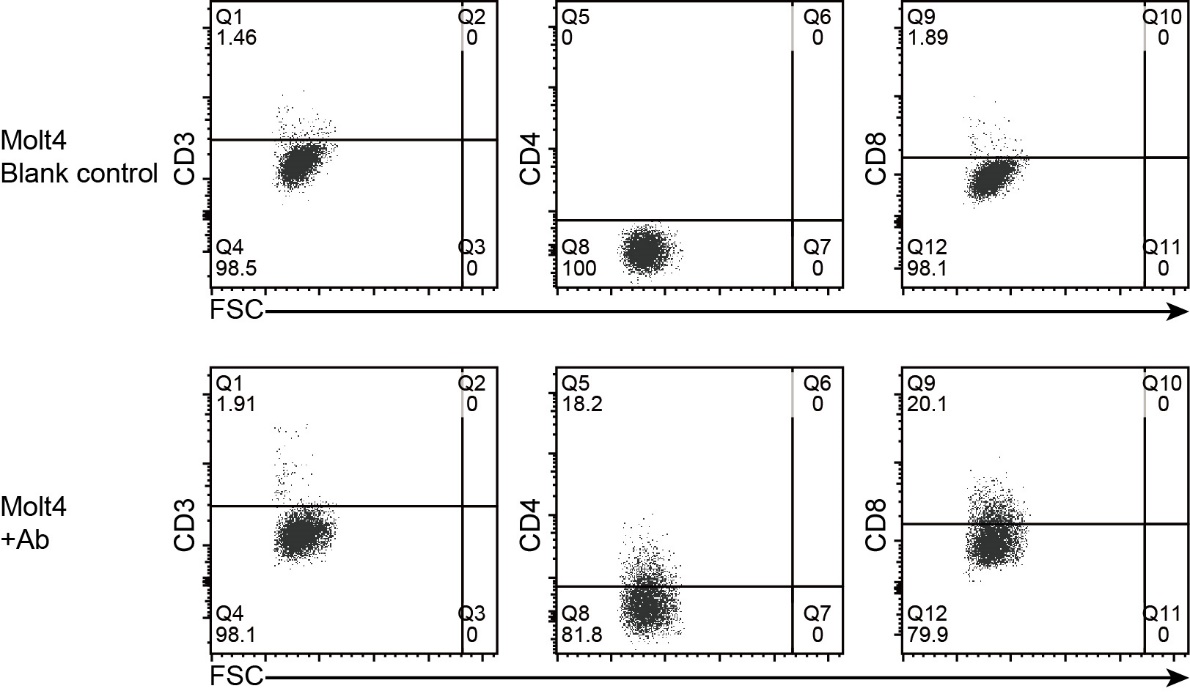


| Figure13. FACS analysis of CD3, CD4 and CD8 in Molt4 |
| --- |
